# Abnormal Decrease of Macrophage ALKBH5 Expression Causes Abnormal Polarization and Inhibits Osteoblast Differentiation

**DOI:** 10.1155/2023/9974098

**Published:** 2023-07-19

**Authors:** Junquan Weng, Haidong Fan, Huijuan Liu, Su Tang, Yuyan Zheng

**Affiliations:** ^1^Department of Stomatology, Shenzhen People's Hospital, Shenzhen 518020, Guangdong, China; ^2^The Second Clinical Medical College, Jinan University, Shenzhen, China; ^3^The First Affiliated Hospital, Southern University of Science and Technology, Shenzhen, China

## Abstract

Peri-implant tissue inflammation is an inflammatory injury that occurs in the soft and hard tissues surrounding the implant and is the main cause of short- or long-term failure of implant prosthetic restorations, which is compounded by bone loss and bone destruction in the alveolar bone of diabetes patients with peri-implantitis. However, the mechanisms underlying the persistence of diabetic peri-implantitis, as well as the essential connections and key molecules that regulate its start and progression, remain unknown. In this study, we discovered that M1 macrophage polarization was abnormally enhanced in diabetic peri-implantitis and influenced the osteogenic differentiation of mesenchymal stem cells. RNA sequencing revealed that ALKBH5 expression was abnormally reduced in diabetic peri-implantitis. Further mechanism study showed that ALKBH5 and its mediated m^6^A can influence osteogenic differentiation, which in turn influences the persistence of diabetic peri-implantitis. Our findings present a new mechanism for the suppression of osteoblast development in diabetic peri-implantitis and a new treatment strategy to promote anabolism by inhibiting ALKBH5.

## 1. Introduction

With the advancement of oral implant technology, implant prosthesis, known as the “third tooth” of humans, has become the preferred option of treatment for repairing defective or missing teeth [1–3]. Peri-implantitis is an inflammation of the connective tissue around the implant caused by bacterial infection and progressive bone resorption and destruction, which is the primary cause of implant prosthesis failure in the short or long term, with bone loss and bone destruction in the alveolar bone of diabetes patients with peri-implantitis being more severe [4, 5]. Since basic blood sugar management and bacterial infection prevention cannot completely control the continuous resorption and destruction of bone tissue around diabetes implants, the implants eventually loosen and come off, causing significant discomfort to diabetic patients [6–8]. Therefore, it is critical in the field of dental implant restoration research to explore the mechanism of diabetic peri-implantitis, identify the main connections and key molecules that govern its development and progression, intervene in it, and discover novel effective therapeutic targets.

Diabetes mellitus is not only a metabolic disease but also a chronic systemic inflammatory disease characterized by a continuous abnormal activation of inflammatory cells, suggesting that glucose (GLU) metabolism disorders may not be the entire mechanism of more severe bone loss and bone destruction in alveolar bone in patients with type II diabetic peri-implantitis [6, 9, 10]. Furthermore, when peri-implantitis is combined with type II diabetes, the immunological inflammatory microenvironment is drastically changed. Due to the periodontal membrane's barrier, there is little information exchange between gingival tissue and alveolar bone under normal conditions. However, numerous recent investigations have revealed that the absence of periodontal membrane in peri-implantitis allows for “information exchange” between peri-implant soft tissue inflammatory cells and alveolar bone mesenchymal stem cells (MSCs) [11]. Notably, it has been shown that when MSCs are adhered to the implant surface for culture, the cells sensitively sense changes in the microenvironment and adapt to it, enabling safe and stable cell proliferation and inducing differentiation into bone cells and promoting angiogenesis [12]. Exploring the more severe etiology of peri-implantitis in type II diabetes is likely to lead to the discovery of novel critical mechanisms and therapeutic options from the standpoint of “interactive dialog” between inflammatory cells and MSCs in peri-implant soft tissues.

Macrophages are one of the most important inflammatory cells that antagonize infection, regulate the immune response, and maintain the stability of the internal environment [13], and are also the main infiltrating cells in the chronic inflammatory response around implants [14]. Functionally, macrophages can be classified into classical pathway-activated (M1), alternative pathway-activated (M2), and intermediate types. M1-type macrophages and M2-type macrophages are in an anti-inflammatory and pro-inflammatory balance during a normal immunological inflammatory response. Recent studies have shown that this balance is disturbed in type II diabetes [15], with continued abnormal activation of macrophages, as evidenced by excessive M1 polarization and restricted M2 polarization, resulting in disruption of the inflammation-suppressing mechanisms and further progression of the disease. Recent studies have shown an increase in the number of macrophages in peri-implantitis and a distinctive M1 polarization profile. Moreover, the M1/M2 polarization imbalance of the macrophage system in type II diabetic patients significantly inhibits the osteogenic differentiation of MSCs, preventing bone regeneration and aggravating peri-implantitis. Therefore, to investigate the role of abnormal macrophage polarization in the bone resorption and destruction of peri-implantitis in type II diabetes and to clarify the specific mechanism is expected to discover a new mechanism for the persistence of peri-implantitis in type II diabetes and to provide new strategies for its prevention and treatment.

## 2. Materials and Methods

### 2.1. Mice Studies

All mice were maintained under specific pathogen-free conditions, and all studies were approved by the ethical review process of Shenzhen People's Hospital. For the establishment of type II diabetes mice model, 4-week-old male C57BL/6J mice were fed with high-sugar chow for 3 weeks continuously, induced by a single intraperitoneal injection of 2% streptozotocin, and fasting GLU was measured by glucometer. Mice with GLU ≥ 16.7 mmol/L were defined as type II diabetic model mice. To introduce peri-implantitis mouse model, 4-week-old male C57BL/6J mice were anesthetized with 1% sodium pentobarbital (0.15 mL/20 g) by intraperitoneal injection, the limbs were fixed in the supine position, the oral cavity and perioral area were disinfected with 1% tincture of iodine, the maxillary right first molar was extracted minimally, and a custom-made titanium self-tapping implant (0.8 × 1.5 mm) was placed after 8 weeks. the end of the suture to induce peri-implantitis. To establish the mouse model of peri-implantitis with type II diabetes, 4-week-old male C57BL/6J mice were first induced type II diabetes for 3 weeks and then subjected to induction for the development of peri-implantitis.

In the mouse model of peri-implantitis with type II diabetes, additional safety precautions were taken, including maintaining specific pathogen-free conditions, obtaining ethical approval, using appropriate anesthesia, practicing sterile techniques, and monitoring the animals' welfare. Type II diabetes was induced first for 3 weeks before the induction of peri-implantitis, ensuring that the animals' physiological conditions were stable and compatible with the study objectives.

### 2.2. Single-Cell Suspension Preparation

To prepare a single-cell suspension of mouse periodontal soft tissue, the soft tissue surrounding the mouse teeth was first dissected and isolated. The isolated tissue was then placed in a centrifuge tube containing phosphate-buffered saline (PBS) to remove impurities, such as blood, that may have been attached to the tissue surface. Subsequently, the tissue was cut into small pieces using sterile scissors and digested with trypsin. After digestion, the tissue was centrifuged, and the supernatant was removed. To ensure the cells were suspended uniformly, PBS with 0.04% bovine serum albumin was added. Finally, the remaining tissue debris and impurities were removed by filtration to obtain a single-cell suspension of mouse periodontal soft tissue. It is important to maintain sterile conditions during the entire process to prevent contamination.

### 2.3. Cell Culture and Cell Transfection

The human mesenchymal stem cell (hMSCs) were obtained from Shanghai Biology Institute (Shanghai, China) and cultured in Dulbecco's Modified Eagle's Medium (DMEM, Gibco, 11885-076) and supplemented with 10% fetal bovine serum (FBS, Gibco, 10270-106) and 1% penicillin-streptomycin (Gibco, 15140122) at a 37°C incubator containing 5% CO_2_.

siRNA targeting ALKBH5 or negative control (NC) was transfected into hMSCs by Lipofectamine™ 2000 transfection reagent (Thermo Fisher Scientific, 11668019). The sequences of si-ALKBH5 and NC were listed as follows: si-ALKBH5-1:5ʹ-AAACUCUACUUGUCCUUCUGAGU-3ʹ; si-ALKBH5-2:5ʹ-ACAAGUACUUCUUCGGCGA-3ʹ; NC: 5ʹ-CAAACTACGGAGTGGACACTCCTCA-3ʹ. Overexpression of ALKBH5 was achieved by transfecting a human-tagged open reading frame clone of ALKBH5 with an empty vector as control.

### 2.4. Flow Cytometry

As stated above, single-cell suspensions of mice soft tissues were obtained. The ratio of M1 and M2 macrophages in mice was measured by flow cytometry (CytoFLEX, Beckman Coulter). Type M1 cells were labeled with antibodies CD68 (leukocyte differentiation antigen 68), CCR2 (chemokine-C receptor-2), and CCR7 (chemokine-C receptor-7), while type M2 cells were labeled with antibodies CD163 (leukocyte differentiation antigen 163), CX3CR1 (chemokine-X3C receptor-1), and CD206 (leukocyte differentiation antigen 206). And the M1/M2 type ratio was calculated.

### 2.5. Immunofluorescence Staining

Mice peri-implant soft tissue specimens were fixed with 4% formaldehyde, permeabilized with 0.2% Triton X-100 for 5 min, blocked with 5% BSA-PBS for 1 hr at room temperature, and incubated overnight with anti-p-MLC2 (p-MLC2S19, 1 : 200 in 5% BSA-PBS) at 4°C. Alexa-488 anti-rabbit secondary antibody (Life Technologies) at a ratio of 1 : 500 was used for 1 hr at room temperature. Cell nuclei were stained with DAPI (Life Technologies). Imaging was performed on a Zeiss LSM 510 Meta confocal microscope (Carl Zeiss) and analyzed using Zen software (Carl Zeiss).

### 2.6. Real-Time Quantitative PCR (qPCR)

Total RNA was obtained using the TRIZOL reagent, followed by reversely transcribed into cDNA using the high-capacity RNA-to-cDNA Kit (Thermo Fisher Scientific, Cat#4387406). Then the cDNA was used for real-time polymerase chain reaction (PCR) according to the instructions provided by the TB Green™ Premix Ex Taq II kit (TaKaRa, RR820B). The amplification procedure involved the following parameters: initial denaturation at 94°C for 30 s, followed by 40 amplification cycles at 94°C for 12 s (denaturation), 58°C for 30 s (annealing), and 72°C for 45 s (extension). The following primers were used: IL-6: reverse primer: ACCACTTCACAAGTCGGAG; forward primer: GAATTGCCATTGCACAACTC; IL1-b: reverse primer: AAGGAGAACCAAGCAACGA; forward primer: GATCCACACTCTCCAGCTG; Tnf: reverse primer: TTCTCATTCCTGCTTGTGG; forward primer: TTGGGAACTTCTCATCCCT; Mrc1: reverse primer: ACACAAATTCAGGGTTCTGG; forward primer: TGATGCTGCTGTTATGTCTC; Arg1: reverse primer: GAACTGAAAGGAAAGTTCCCA; forward primer: AATGTACACGATGTCTTTGGC; IL-10: reverse primer: TGGCCCAGAAATCAAGGAG; forward primer: GAGAAATCGATGACAGCGC; Runx2: reverse primer: ATGATGAGAACTACTCCGCC; forward primer: GTGAAACTCTTGCCTCGTC; OSX: reverse primer: CTGCTTGAGGAAGAAGCTC; forward primer: TTCTTTGTGCCTCCTTTCC; Col1a1: reverse primer: ATACTGGTGTTAAAGGTGATGC; forward primer: CACCAACGTTACCAATGGG; GAPDH: reverse primer: ACTCTTCCACCTTCGATGC; forward primer: CCGTATTCATTGTCATACCAGG.

### 2.7. Immunoblotting

Total protein was extracted using radioimmunoprecipitation assay buffer lysis buffer, followed by separated by 10% sodium dodecyl sulfate polyacrylamide gel electrophoresis, transferred to polyvinylidene fluoride membrane, blocked in 5% skimmed milk at room temperature for 1.5 hr. Subsequently, primary antibody (Anti-ALKBH5, SAB1407587, Sigma-Aldrich) was added overnight at 4°C. On the next day, the membrane was incubated with horseradish peroxidase-labeled secondary antibody (7076s, Cell Signaling Technology) at room temperature for 2 hr. For detection, ECL detection system was used.

### 2.8. Alizarin Red S Staining

The cells were washed three times with PBS and fixed in 75% alcohol for 30 min. The fixed cells were stained in 0.1% Alizarin Red-Tris–HCl solution (pH 8.2) for 10 min; after the staining, the cells were rinsed rapidly with 1% acetic acid solution and dehydrated with different concentrations of ethanol in turn; the degree of osteogenic differentiation was analyzed after inverted microscopic photography.

### 2.9. Dot Blot

Briefly, equal amounts of RNA were denatured at 95°C for 5 min, followed immediately cooling at 4°C for 1 min. Afterward, RNA was added to Amersham Hybond N+ membranes and cross-linked for 30 s on a Stratalinker 2400 UV cross-linker with 2,000 mJ. The membranes were blocked with 5% skim milk for 1 hr at room temperature, and then the anti-m^6^A antibody (anti-m^6^A antibody, Abcam) was incubated overnight at 4°C. After several washes with 0.1% phosphate buffered saline with Tween-20, the membranes were incubated with horseradish peroxidase-coupled secondary antibody (7074s, Cell Signaling Technology) at room temperature for 1 hr. The membranes were developed with a Chemiluminescence Imaging System (Tanon 5200 SF, China).

### 2.10. RNA-Sequencing

RNA samples were extracted from control as well as diabetic mice from peri-implant soft tissue. An amount of 1 *µ*g of total RNA from each sample was analyzed using the Illumina HiSeq 2500 platform. The Illumina Tru-seq RNA sample prep kit was used to prepare libraries. Paired reads were aligned to the mouse mm10 genome using STAR (v2.5.2b). Differential gene expression analysis was performed using the R package DESeq2. Genes with absolute Log2FC values greater than or equal to 1 and *p*-values less than 0.01 were differentially expressed genes (DEGs). GO and KEGG enrichment analysis was performed using the DAVID function for the list of genes with estimated fold changes in DESeq2 ranked from high to low.

### 2.11. Statistical Analysis

Statistical analyses were performed using GraphPad Prism 8 software, and *p* < 0.05 was considered statistically significant. Data are expressed as mean values ± SD unless otherwise stated. Statistical comparisons were performed using unpaired Student's *t*-test and one-way (analysis of variance, comparison of three or more groups with one independent variable). Significance was defined as  ^*∗*^*p* < 0.05.

## 3. Results

### 3.1. Macrophage M1/M2 Polarization Imbalance in Type II Diabetic Mellitus

**Figure 1:**
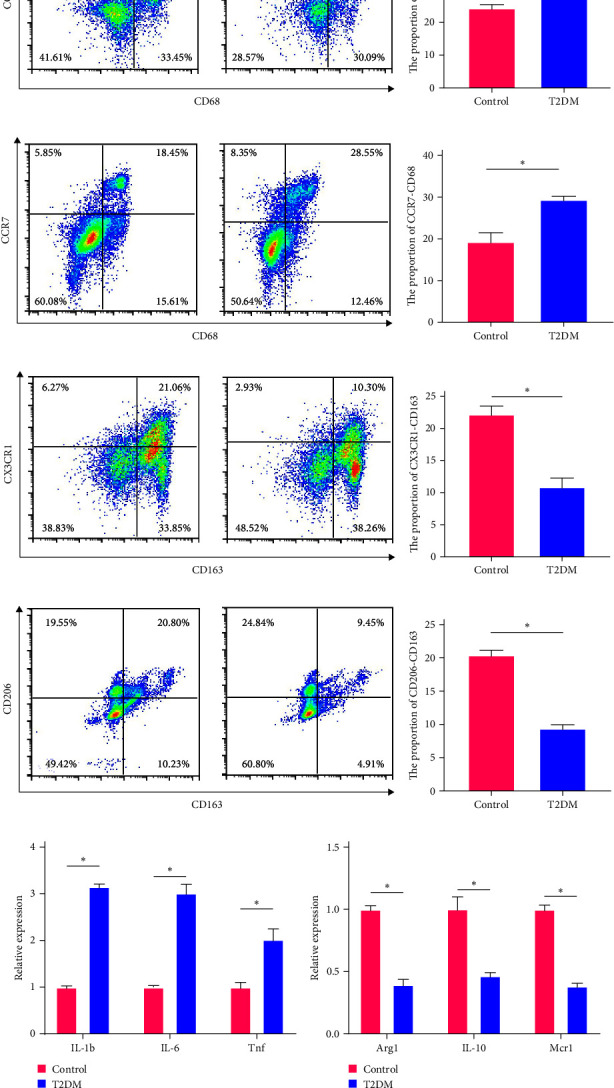
Macrophage M1/M2 polarization imbalance in type II diabetes. (a) The percentages of M1 type macrophage (CD68-CCR2) in type II diabetic mice were analyzed by flow cytometry. (b) Quantification of percentages of M1 type macrophage (CD68-CCR2) in type II diabetic mice. (c) The percentages of M1 type macrophage (CD68-CCR7) in type II diabetic mice were analyzed by flow cytometry. (d) Quantification of percentages of M1 type macrophage (CD68-CCR7) in type II diabetic mice. (e) The percentages of M2 type macrophage (CD163-CX3CR1) in type II diabetic mice were analyzed by flow cytometry. (f) Quantification of percentages of M2 type macrophage (CD163- CX3CR1) in type II diabetic mice. (g) The percentages of M2 type macrophage (CD163-CD206) in type II diabetic mice were analyzed by flow cytometry. (h) Quantification of percentages of M2 type macrophage (CD163-CD206) in type II diabetic mice. (i) qPCR analysis of IL-1b, IL-6, and Tnf mRNA levels in type II diabetic mice. (j) qPCR analysis of Arg1, IL-10, and Mcr1 mRNA levels in type II diabetic mice. Significance was defined as  ^*∗*^*p* < 0.05.

In this study, we aimed to investigate the impact of type II diabetes mellitus on macrophage polarization and its effects on peri-implantitis. We observed a significant alteration in macrophage polarization in the soft tissues of diabetic mice. Specifically, the ratio of M1-type macrophages was found to be significantly increased, while the ratio of M2-type macrophages was decreased compared to the control group (Figure 1(a)–1(h)). This shift in macrophage polarization was further supported by the analysis of macrophage-related mRNA expression in peri-implant soft tissues using qPCR. We observed elevated expression levels of M1-type polarized macrophage markers, including IL-1b, IL-6, and Tnf, indicating an inflammatory phenotype. In contrast, the expression levels of M2-type polarized macrophage markers, such as Arg1, IL-10, and Mrc1, were decreased in type II diabetic mice (Figures 1(i) and 1(j)). These findings suggest an imbalance in macrophage polarization towards a pro-inflammatory state in the peri-implant soft tissues of diabetic mice. Based on these results, we propose that the increased infiltration of M1-type macrophages in the peri-implant soft tissue under the diabetic condition disrupts the osteogenic microenvironment of alveolar bone MSCs. This dysregulated microenvironment may contribute to more severe bone destruction in peri-implantitis combined with type II diabetes.

### 3.2. Peri-Implantitis Combined with Type II Diabetes Has a Significant Macrophage M1/M2 Polarization Imbalance

**Figure 2:**
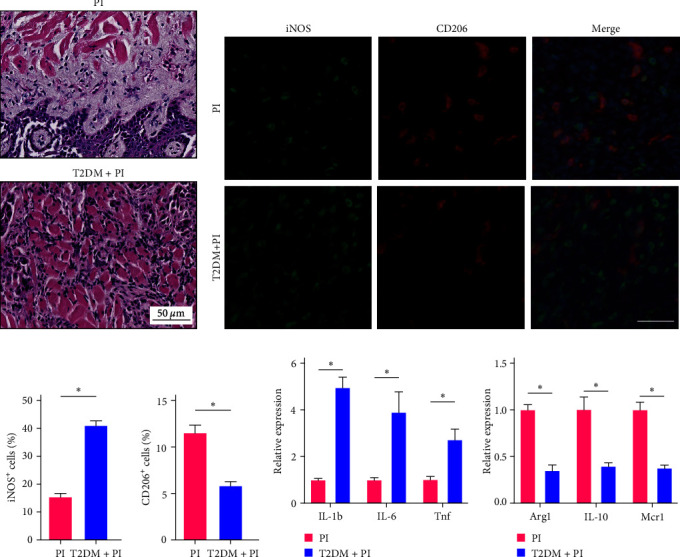
Peri-implantitis combined with type II diabetes is associated with a large imbalance in macrophage M1/M2 polarization. (a) H&E staining of soft tissue from peri-implantitis in type II diabetic and control mice. Scale bar, 50 *μ*m. (b) Representative images of iNOS (green), CD206 (red), and DAPI nuclear staining (blue) soft tissue from peri-implantitis in type II diabetic and control mice. Scale bar, 50 *μ*m. (c and d) Quantification of percentages of iNOS^+^ (c) and CD206^+^ (d) cells in different treatment groups. (e) IL-1b, IL-6, and Tnf expression were examined by qPCR and normalized to GAPDH expression. (f) Arg1, IL-10, and Mcr1 expression were examined by qPCR and normalized to GAPDH expression. Significance was defined as  ^*∗*^*p* < 0.05.

We observed a significant increase in inflammatory infiltration in the peri-implantitis combined with type II diabetes mice compared to the control group following the induction of peri-implantitis (Figure 2(a)). Immunofluorescence staining of macrophage markers in the peri-implant inflammatory tissues further confirmed an excessive M1 polarization and a restricted M2 polarization in the peri-implantitis combined with type II diabetes group (Figure 2(b)–2(d)). This imbalance in macrophage polarization disrupted the inhibitory mechanism of inflammation, leading to the progression of the disease. Moreover, we analyzed the expression of specific macrophage-related mRNA in the peri-implant soft tissues using qPCR. We found that the expression levels of M1 polarized macrophage-related markers, including IL-1b, IL-6, and Tnf, were significantly enhanced in the peri-implantitis coupled with type II diabetes group (Figure 2(e)). Conversely, the expression levels of M2 polarized macrophage-related markers, such as Arg1, IL-10, and Mrc1, were significantly lowered in the same group (Figure 2(f)). These results further support an intensified inflammatory response and a compromised regulatory mechanism in peri-implantitis combined with type II diabetes. Taken together, our findings highlight the dysregulated macrophage polarization and altered expression of macrophage-related markers in the peri-implant inflammatory tissues of type II diabetes mice.

### 3.3. Peri-Implantitis with Diabetes Mice Downregulate ALKBH5 Expression and Osteogenic Differentiation

**Figure 3:**
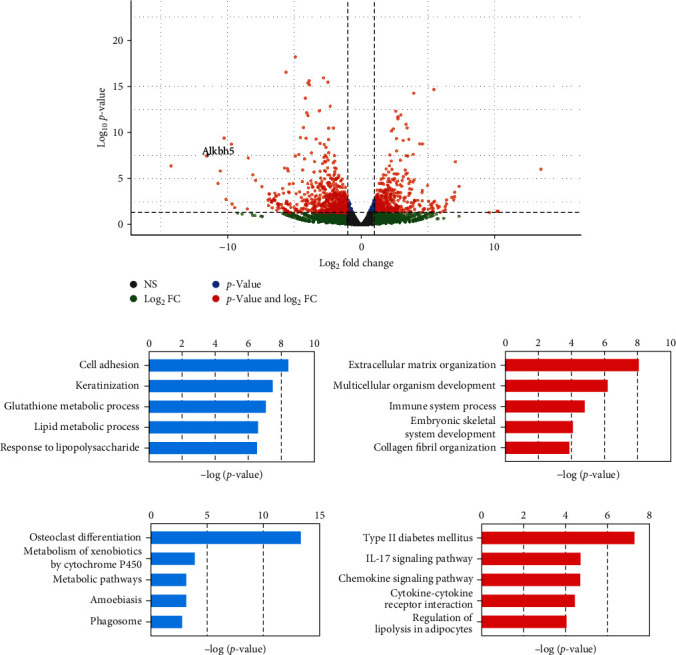
Diabetes mice with peri-implantitis reduce ALKBH5 expression and osteogenic differentiation. (a) Volcano plot for genes differentially expressed between diabetic and non-diabetic mice with peri-implantitis. (b) Gene ontology (GO) of biological processes (BP) analysis of the downregulated DEGs. (c) Gene ontology (GO) of biological processes (BP) analysis of the upregulated DEGs. (d) KEGG analysis of the downregulated DEGs. (e) KEGG analysis of the upregulated DEGs.

To assess the impact of diabetes on peri-implantitis, we conducted transcriptome analysis in mice. A comparison between diabetic and non-diabetic mice with peri-implantitis revealed significant alterations in gene expression. Specifically, we identified 285 upregulated and 420 downregulated transcripts in the diabetic infection group (fold change > 2, *p*-value < 0.01) (Figure 3(a)). Notably, genes involved in cell adhesion, keratinization, and glutathione metabolic process were found to be downregulated, while genes related to extracellular matrix organization and multicellular organism development were upregulated in peri-implantitis combined with type II diabetes (Figures 3(b) and 3(c)). Pathway analysis of these DEGs provided further insights. We observed a significant downregulation of the Osteoclast differentiation pathway in diabetic infection, whereas pathways such as type II diabetes mellitus, IL-17 signaling, and chemokine signaling were upregulated (Figures 3(d) and 3(e)). Importantly, ALKBH5 expression was significantly downregulated (log fold change = −11.56) in the peri-implantitis combined with type II diabetes group (Figure 3(a)). These findings suggest that ALKBH5 and its mediated m^6^A demethylation may play a crucial role in regulating osteogenic differentiation in peri-implantitis combined with type II diabetes mellitus. The downregulation of ALKBH5 expression highlights its potential significance in modulating osteogenic differentiation in this pathological context.

### 3.4. ALKBH5 Promotes Osteogenic Differentiation of hMSCs

**Figure 4:**
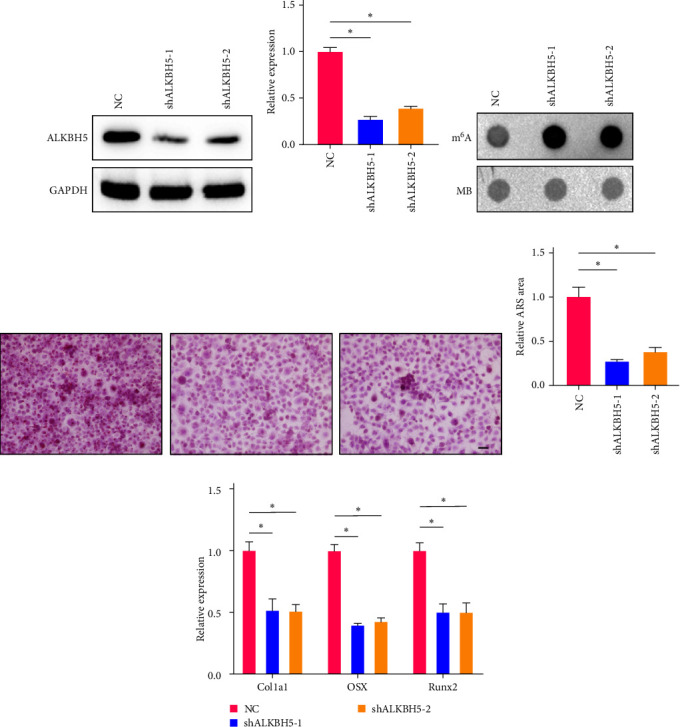
ALKBH5 promotes hMSC osteogenic differentiation. (a) ALKBH5 levels of cell transfected with or without sh-ALKBH5 were detected by Western blot (WB). (b) ALKBH5 mRNA levels of cell transfected with or without sh-ALKBH5 were detected by qPCR. (c) Dot-blot analysis of RNA m^6^A modification levels of cell transfected with or without sh-ALKBH5. MB, methyl blue. (d) hMSC osteogenic differentiation analyzed by Alizarin red–S. Scale bar, 50 *μ*m. (e) Quantification of Alizarin red–S staining area in different groups. (f) Col1a1, OSX, and Runx2 mRNA levels of cell transfected with or without sh-ALKBH5 were detected by qPCR. Significance was defined as  ^*∗*^*p* < 0.05.

To investigate the impact of ALKBH5 on osteogenic differentiation in peri-implantitis combined with type II diabetes, we employed siRNAs to downregulate ALKBH5 expression in hMSC cells. Following a 48 hr transfection, ALKBH5 expression was assessed using Western blotting and qPCR (Figures 4(a) and 4(b)), confirming successful knockdown. Interestingly, we observed an increase in the m^6^A level of the cells upon ALKBH5 suppression (Figure 4(c)), indicating the involvement of ALKBH5 in m^6^A demethylation. Subsequently, we conducted an osteogenic differentiation assay to assess the impact of ALKBH5 knockdown. Remarkably, we observed a decrease in the osteogenic differentiation ability of cells lacking ALKBH5 (Figures 4(d) and 4(e)). Moreover, the expression of osteogenic differentiation markers (Col1a1, OSX, Runx2) was significantly suppressed upon ALKBH5 knockdown (Figure 4(f)), suggesting a hindered state of osteoblast differentiation. In summary, our findings demonstrate that silencing ALKBH5 inhibits osteoblast differentiation. The elevated m^6^A level and impaired expression of osteogenic markers further support the crucial role of ALKBH5 in regulating the osteogenic differentiation process in peri-implantitis combined with type II diabetes.

### 3.5. Overexpression of ALKBH5 Inhibits Osteogenic Differentiation of hMSCs

**Figure 5:**
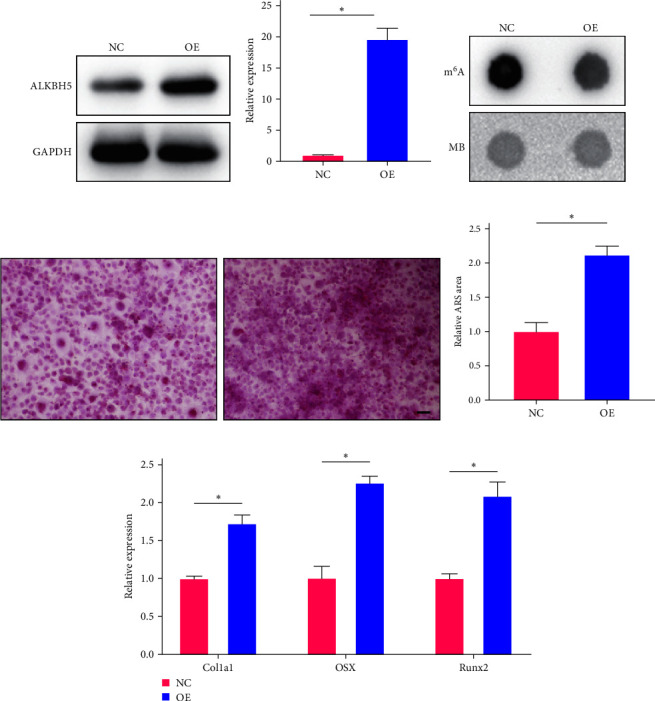
ALKBH5 overexpression impairs hMSC osteogenic differentiation. (a) WB was used to detect the ALKBH5 expression in hMSCs with or without ALKBH5 overexpression. (b) qPCR was used to detect the ALKBH5 mRNA level in hMSCs with or without ALKBH5 overexpression. (c) Dot-blot analysis of RNA m^6^A modification levels of cells with or without ALKBH5 overexpression. MB, methyl blue. (d) hMSC osteogenic differentiation analyzed by Alizarin red–S. Scale bar, 50 *μ*m. (e) Quantification of Alizarin red–S staining area in different groups. (f) Col1a1, OSX, and Runx2 mRNA levels of cells with or without ALKBH5 overexpression were detected by qPCR. Significance was defined as  ^*∗*^*p* < 0.05.

In order to assess the impact of ALKBH5 overexpression on osteogenic differentiation, we employed a lentiviral transduction system to introduce ALKBH5 into hMSC cells. The successful overexpression of ALKBH5 was confirmed through Western blotting and PCR analysis (Figures 5(a) and 5(b)). Notably, the overexpression of ALKBH5 resulted in a significant reduction in cellular m^6^A levels (Figure 5(c)), indicating its role in m^6^A demethylation. Furthermore, we evaluated the effect of ALKBH5 overexpression on osteogenic differentiation. Remarkably, ALKBH5 overexpression significantly enhanced the osteogenic differentiation ability of hMSCs, as evidenced by increased mineralization and enhanced expression of osteogenic markers (Figure 5(d)–5(f)). These findings suggest that ALKBH5 overexpression promotes the osteogenic differentiation potential of hMSCs.

## 4. Discussion

In this study, we determined whether ALKBH5 and its induced m^6^A demethylation can play a role in osteogenesis in a macrophage microenvironment and the potential mechanism. Our study showed that M1 macrophage polarization is aberrant in diabetic peri-implantitis, which impairs MSC osteogenic differentiation. We subsequently found that ALKBH5-mediated m^6^A demethylation modifications can regulate the osteogenic differentiation of MSCs, corresponding to the transition from tissue regeneration to an inflammatory state.

The immune system involves interactions with immune cells that can produce various cytokines related to inflammation and alter the microstructure of bone [16, 17]. Among these cells, macrophages play a crucial role in immune regulation and bone homeostasis. There are two main polarization states of macrophages, classically activated macrophages (M1) and alternatively activated macrophages (M2). For an in-depth understanding of the connection between macrophages and peri-implantitis, we explored the state of macrophages during diabetic peri-implantitis. Previous studies have revealed that in type II diabetes, macrophages are persistently and abnormally active, with hyperpolarized M1 and limited M2 polarization [15, 18]. Consistently, we found that M1 macrophages were significantly higher than M2 macrophages in human and mouse models of diabetic peri-implantitis.

It is widely believed that diverse macrophage polarization states contribute to different bone metabolism disorders [19, 20]. Previous study demonstrated that induction of M2 polarization promoted osteogenic differentiation [21, 22]. M2 macrophage extracellular vesicles increased osteoinductive gene expression in MSCs [23]. To elucidate the molecular mechanisms underlying this difference in macrophage status, we induced peri-implantitis in control as well as type II diabetic mice and performed RNA-seq. The results revealed a reduced level of osteoblast differentiation and an increased level of inflammatory activation in diabetic peri-implantitis compared to control group, confirming that osteoblast differentiation is restricted in diabetic peri-implantitis. The osteogenic process was hindered in type II diabetes-induced inflammatory milieu, boosting the generation of pro-inflammatory cytokines. Furthermore, ALKBH5 expression was found to be significantly decreased in diabetic peri-implantitis, indicating a strong link between ALKBH5 and osteoblast development and inflammation. The overexpression of ALKBH5 has been shown to promote the osteogenic differentiation of MSCs, whereas its deletion has been found to inhibit this process [24]. Therefore, ALKBH5 was selected as a starting point for our study in order to investigate its role in the osteogenic differentiation process in diabetic peri-implantitis.

MSCs are the most extensively studied stem cells and have been intensively studied for clinical translation over the last decades [25–27]. MSCs are employed in the therapy of bone disorders because they can differentiate into osteoblasts to conform to bone creation and repair as pluripotent stem cells [28]. Therefore, we hypothesized that external interventions that direct MSCs differentiation are promising approaches for the treatment of diabetic peri-implantitis. To explore the molecular mechanisms underlying the effects of ALKBH5 and its mediated m^6^A demethylation modifications on osteoblast differentiation under inflammatory conditions, we evaluated m^6^A level in ALKBH5 knockdown cells. As shown in the data, m^6^A levels rose in ALKBH5 knockdown cells, whereas osteoblast differentiation increased. Interestingly, this elevation in m6A levels was accompanied by an enhancement in osteoblast differentiation, suggesting a positive correlation between m^6^A modifications and osteoblast development. Conversely, in ALKBH5 overexpressing cells, the level of m^6^A decreased, indicating a reduction in m^6^A modifications. Remarkably, this decrease in m^6^A levels was associated with a restriction in the differentiation capacity of osteoblasts, implying a negative impact of reduced m^6^A modifications on osteoblast development. When translating the preclinical findings to clinical practice, targeting ALKBH5 and m^6^A demethylation emerges as a promising strategy to enhance osteoblast differentiation and alleviate inflammation in peri-implantitis patients. This approach holds potential for improving treatment outcomes and gaining deeper insights into the underlying disease mechanisms.

In diabetic peri-implantitis, the decreased expression of macrophage ALKBH5 is linked to abnormal cell polarization and inhibition of osteoblast differentiation. These dysfunctions in osteoblast development have implications beyond the implant site. Osteoblasts are vital for maintaining bone density and integrity. Deformations in osteoblasts can lead to significant bone density changes, impacting dental health. Reduced bone density resulting from osteoblast deformations can contribute to major tooth problems. As bone provides support for teeth, disruptions in density weaken tooth structure, leading to loosening, increased vulnerability to dental caries, and potential tooth loss. Considering the impact of osteoblast deformations on bone density and their connection to tooth problems, our study emphasizes the need to address these abnormalities in diabetic peri-implantitis. Understanding the mechanisms and developing strategies to enhance osteoblast development could potentially improve dental outcomes in affected individuals.

Limitations of our study include the reliance on in vitro experiments, necessitating further validation using animal models or clinical samples. Additionally, the role of other factors and pathways in addition to ALKBH5 and m^6^A modifications should be considered. Future research can focus on investigating downstream signaling pathways and molecular targets influenced by ALKBH5-mediated m^6^A demethylation. Exploring potential crosstalk between ALKBH5 and other epigenetic modifications, such as DNA methylation or histone modifications, can reveal novel regulatory networks in osteoblast development. Furthermore, investigating the therapeutic potential of targeting ALKBH5 and m^6^A modifications in treating diabetic peri-implantitis and promoting osteoblast anabolism is a promising direction for future studies. When discussing our findings, it is important to consider other factors that can impact peri-implantitis and osteoblast differentiation alongside ALKBH5 and m^6^A demethylation. Dental diseases, metabolic disorders like diabetes and obesity, drug treatments, and factors like infection and trauma can all disrupt normal bone metabolism. Understanding these influences is crucial for a comprehensive understanding of peri-implantitis and osteoblast differentiation. Future research should explore these interactions to better manage bone health issues related to peri-implantitis.

In summary, our findings provide compelling evidence that in diabetic peri-implantitis, M1 macrophage polarization is increased, ALKBH5 expression is decreased, and m^6^A modification is elevated, thereby limiting osteoblast development. These observations highlight the critical role of m^6^A modifications mediated by ALKBH5 in regulating osteoblast differentiation under inflammatory conditions.

## Data Availability

The data used to support the findings of this study are included within the article.
